# Genome-Wide Regulation of Electroacupuncture and Treadmill Exercise on Diet-Induced Obese Rats

**DOI:** 10.1155/2020/8764507

**Published:** 2020-09-24

**Authors:** Yanji Zhang, Jia Li, Dan Wei, Guoyan Mo, Chaochao Yu, Lihua Wang, Yue Zhuo, Kou Xu, Yingrong Zhang, Yixuan Xue, Wei Huang, Zhongyu Zhou

**Affiliations:** ^1^College of Acupuncture and Orthopedics, Hubei University of Chinese Medicine, Hubei Provincial Collaborative Innovation Center of Preventive Treatment by Acupuncture and Moxibustion, Wuhan, China; ^2^Department of Acupuncture, Hubei Provincial Hospital of Traditional Chinese Medicine, Wuhan, China; ^3^Affiliated Hospital of Hubei University of Chinese Medicine, Wuhan, China; ^4^China Key Laboratory of TCM Resource and Prescription, Hubei University of Chinese Medicine, Ministry of Education, Wuhan, China; ^5^Department of Tuina, Shenzhen Traditional Chinese Medicine Hospital, Shenzhen, China

## Abstract

Acupuncture has been widely used for obesity treatment, but its mechanism is still unclear. To investigate the molecular mechanisms, we applied electroacupuncture (EA) at the Zusanli (ST36) acupoint and treadmill exercise (TE) in a diet-induced obese (DIO) rat model and used RNA sequencing (RNA-seq) to identify molecular consequences. Forty Sprague-Dawley male rats were selected and randomly divided into five groups: control (C), DIO model (M), EA, TE, and EA + TE groups. According to the results, acupuncture reduced body weight and the ratio of retroperitoneal white adipose tissue (retro-WAT) to body weight. Total RNA was extracted from the retro-WAT from five groups for RNA-seq. Differentially expressed genes (DEG) analysis showed that there were obvious differences among the four comparisons of C vs. M, M vs. EA, M vs. TE, and M vs. EA + TE, followed by 1383, 913, 3324, and 2794 DE genes. Gene ontology (GO) term enrichment analysis and Kyoto Encyclopedia of Genes and Genomes pathway enrichment analysis were performed to further classify the DEGs. Several GO terms were commonly significantly enriched in both M vs. TE and M vs. EA, such as myofibril and muscle contraction. In addition, some pathways were regulated by EA and TE, such as the peroxisome proliferator activated receptor signaling pathway and calcium signaling pathway. This study is the first to compare and analyze the differences in gene expression profiles in the retro-WAT of rats in different groups, which provide a clue for further investigation into the molecular mechanisms of obesity treatment by EA and TE.

## 1. Introduction

In recent years, the number of obese persons has increased rapidly. Approximately 1.9 billion adults are overweight and 600 million are obese worldwide [1]. According to formal research, obesity is associated with many health concerns, such as type 2 diabetes mellitus (T2DM), cardiovascular disease, and cancer [2, 3]. The incidence of cardiovascular events in patients with excessive waist circumference is 2.1 times that in patients with normal waist circumference [4]. For every 11 cm increase in waist circumference, the risk of obesity-related cancer increases by 13% [5]. According to a report by the Organization for Economic Cooperation and Development (OECD), 70% of diabetes treatment costs, 23% of cardiovascular disease treatment costs, and 9% of cancer treatment costs are caused by obesity. On average, the costs of treating diseases caused by obesity account for an average of 8.4% of total health care expenditure in OECD countries [6].

There are different methods to treat obesity, such as lifestyle modification, drugs, surgery, and complication therapy. However, most of the current antiobesity drugs have failed to achieve adequate weight control in patients due to side effects [7, 8]. Lifestyle modification, especially exercise, is an important component for the treatment of obesity [9]. Physical exercise can reduce percentage body fat and cardiometabolic risk markers and increase lean body mass and leg muscle strength [10]. Even after bariatric surgery, physical training is still necessary to improve aerobic capacity and preserve lean mass [11, 12]. Previous studies have shown that accumulating 150 min/week of moderate physical activity is effective in preventing weight gain; ≥250 min/week of moderate physical activity can achieve clinically significant weight loss [13].

In recent years, acupuncture has been widely used for obesity treatment. Several meta-analyses have shown that electroacupuncture (EA), auricular acupoints, and acupoint embedding can effectively treat obesity, and it is not a placebo effect [14–16]. The effect of acupuncture on weight loss is related to various mechanisms, such as regulation of the endocrine system, promotion of digestion, attenuation of oxidative stress, and alteration of bacterial diversity [17–19]. Our previous work showed a reduction in the body weight of diet-induced obese (DIO) rats treated with EA on Zusanli (ST36) and Tianshu (ST25) acupoints, along with a significant decrease in their plasma triglyceride (TG) and total cholesterol (TC) levels. Additionally, acupuncture can upregulate PGC-1*α*/UCP-1 signaling in white adipose tissue (WAT), suggesting the efficacy of EA in promoting the browning of WAT [20]. There is no doubt that a single factor may not be enough to explain the beneficial effects of acupuncture against simple obesity. Thus, is there a special mechanism for acupuncture antiobesity? What are the commonalities and differences between EA and exercise in the molecular mechanism of weight loss?

Therefore, first, we applied EA treatment and treadmill exercise (TE) treatment in DIO rats to evaluate the effectiveness of EA and TE. Second, we explored the antiobesity mechanism of EA and TE at the molecular level of the transcriptome in the WAT of DIO rats by RNA sequencing (RNA-seq). Our data suggest a difference between the control group (C), DIO model group (M), EA group, TE group, and EA + TE group. The differences existed in gene expression level, GO categories, and pathway categories, which contribute to our knowledge of the effect of EA on the WAT of DIO rats at the molecular level.

## 2. Materials and Methods

### 2.1. Animals and Grouping

Seventy 4-week-old SD male rats were obtained from the Animal Experimental Center of China Three Gorges University (experimental animal production license number: SCXK [Hubei] 2017–0012). All rats were raised in individually ventilated cages under a 12 h light/dark cycle (light on 8 : 00am to 8 : 00pm) with free access to water and chow food; ambient temperature and relative humidity were maintained at 21 ± 2°C and 50% ± 10%. All animal treatments were approved by the Animal Ethics Committee of the Hubei Provincial Hospital of Traditional Chinese Medicine (No. 2018012).

After one week of adaptation, the rats were randomly divided into two groups as follows: the basal diet group (*n* = 8; 10% fat, Research Diets No. D12450 B) and high-fat diet group (*n* = 62; 60% fat, Research Diets No. D12492). After 12 weeks on the high-fat diet, the body weight of rats fed a high-fat diet tended to increase 20% more than rats in the control group and were defined as DIO rats. We then divided the 32 DIO rats into the following four groups. (1) EA group was administered EA at Zusanli (ST36) for eight weeks. (2) The TE group was applied with TE for 8 weeks. (3) The EA + TE group was treated with both EA and TE. (4) Model group (M) was not treated.

### 2.2. EA Intervention

**Figure 1:**
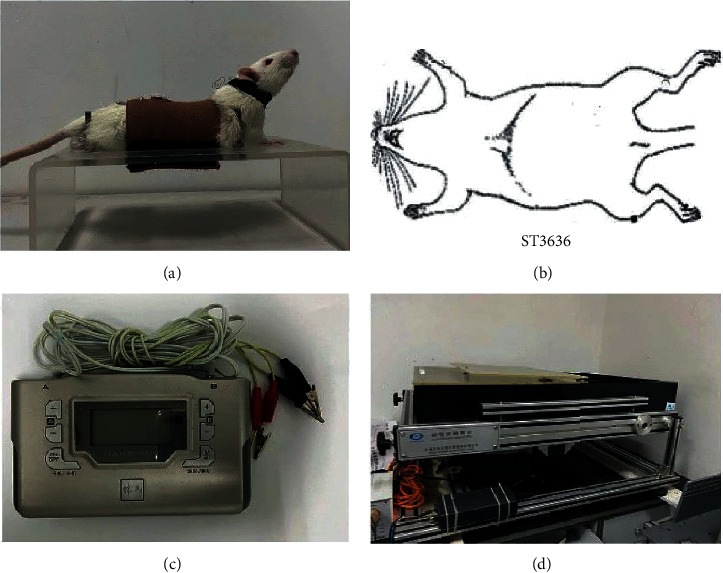
Electroacupuncture and treadmill exercise instrument and diagram of acupoint Zusanli (ST36). (a) Holder for fixation of rat to decrease unconsciousness and discomfort. (b) Schematic diagram of Zusanli (ST36). (c) Han's acupoint nerve stimulator, HANS-200A. (d) Treadmill exercise instrument.

Rats were fixed on a homemade holder instrument (Figure 1(a)) for EA operation. A sterile acupuncture needle was inserted into rats in the EA and EA + TE groups (diameter: 0.35 mm, length: 40 mm; Suzhou Medical Supplies Factory Co., Ltd., Suzhou, China) at Zusanli (ST36, located at the anterior tibial muscle and about 3 mm below the knee joint) acupoint (Figure 1(b)) at a depth of 3 mm. Then, an electrical current was provided to the needles through an electrical stimulator for 30 min, with a stimulus isolation unit (Han's acupoint nerve stimulator, HANS-200A, Beijing, China, Figure 1(c)) at a frequency of 2/15 Hz and an intensity level of 1 mA. The EA treatment was performed once a day, five times per week for eight weeks. Rats in the model group were fixed as the EA group but were not given any treatment.

### 2.3. TE Intervention

According to the method by Bedford [21], the load was set at 16 m/min and 60 min/d, equivalent to 58% of the maximum oxygen uptake in rats (Figure 1(d)). TE was performed five days per week for a total of eight weeks. During the adaptive training (first week), the load was started from 6 m/min, 10 min/time and then progressively increased at a rate of 2 m/min for 10 min. Rats that were not suitable for TE or did not receive the exercise for a long time due to injury were excluded. The actual exercise was started in the second week.

### 2.4. Tissue Sampling

At the end of the treatment, all rats were fasted overnight. Each rat was euthanized with intravenous injections of high-dose pentobarbital. The retro-WAT was washed with cold normal saline, immediately frozen in liquid nitrogen, and stored at −80°C for RNA extraction.

### 2.5. RNA Extraction and RNA-Sequencing Analysis

Total RNA of retro-WAT was extracted using an RNeasy Mini Kit (Qiagen, Hilden, Germany), and RNA quality was assessed using an Agilent 2100 Bioanalyzer (Agilent Technologies). The library preparation was conducted using an MGI EasyTM mRNA Library Prep Kit (BGI, Inc., Wuhan, China) following the manufacturer's instructions. cDNA library construction and sequencing were performed using the BGISEQ-500 platform and repeated 10 times. We identified differentially expressed genes (DEGs) between samples according to the default criterion of a false discovery rate <0.05. Clustering analysis and functional annotation were performed. The reference genes were from Rattus_norvegicus-NCBI_Rnor_6.0 (https://www.ncbi.nlm.nih.gov/assembly/GCF_000001895.5).

### 2.6. Statistics Analysis

For statistical purposes, all data were analyzed using Graph Pad Prism 5.0 and expressed as mean ± standard error of the mean. For group comparison, a one-way analysis of variance was used. A *p* value of less than 0.05 was considered statistically significant.

## 3. Results

### 3.1. EA + TE Reduces Body Weight and WAT Weight in DIO Rats

**Figure 2:**
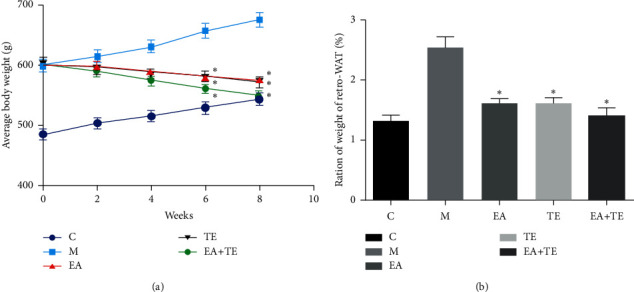
EA + TE reduces body weight and white adipose tissue weight in DIO rats. (a) Observation of body weight in the four groups, *n* = 8 in each group, compared to the model group, *p* < 0.05. (b) Ratio of retro white adipose tissue to mouse body weight, *n* = 8 in each group, compared to the model group, *p* < 0.05.

During the eight weeks of intervention, the body weight of the rats was recorded weekly. Weight loss existed in all treatment groups after eight weeks of intervention, of which weight loss in the EA + TE group was the greatest (*p* < 0.05) (Figure 2(a)).

Meanwhile, we harvested the retro white adipose tissue (retro-WAT) from each group. The ratio of retro-WAT to body weight was calculated. The results indicated that, in rats that were fed a high-fat diet, the ratio was significantly higher than that in rats fed a normal diet. We found that EA and TE significantly decreased the ratio, of which the ratio loss in the EA + TE group was the greatest (*p* < 0.05) (Figure 2(b)).

### 3.2. EA + TE Treatment Changed Genome-Wide Expression Profiles of Retro-WAT in DIO Rats

Total RNA was extracted from the retro-WAT of rats in different groups and treated with DNase I. The 28S/18S and RIN/RQN values in all groups were all above the pass criteria (Table 1). The results indicate that RNA purity and integrity are suitable for RNA-seq experiments.

#### 3.2.1. Genome-Wide Analysis of the DIO Rat

To elucidate the molecular mechanism of diet-induced obesity and the effects of EA plus TE, the DEGs between different groups were identified and analyzed. RNA-seq for retro-WAT was performed using high-throughput sequencing. Compared to the control group, 1383 genes were differentially expressed in the retro-WAT in the model group, of which 79.8% (1104) of the genes were upregulated and 20.2% (279) of the genes were downregulated (Table 2).

#### 3.2.2. EA Treatment Reversed the Abnormal Gene Expression in DIO Rats

**Figure 3:**
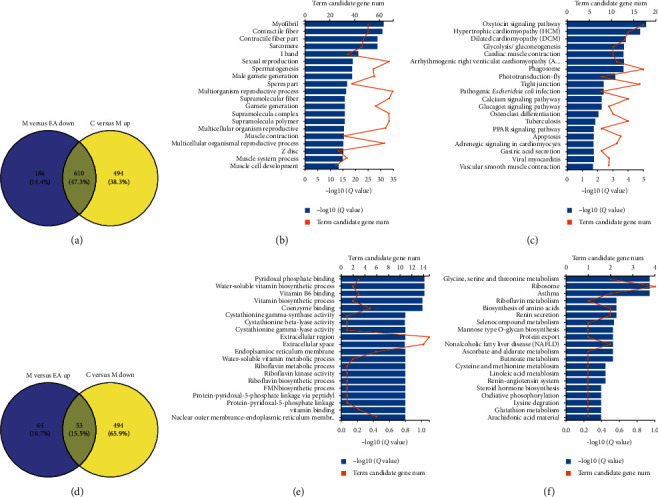
EA treatment reversed the abnormal gene expressions in DIO rats. (a) Purple circle represents number of downregulated DE genes between group M and group EA; yellow circle stands for number of upregulated DE genes between group M and group C. (b) GO annotation results of 610 DEGs in retro-WAT. (c) KEGG pathway for the 610 DEGs. (d) Purple circle represents number of upregulated DE genes between group M and group EA; yellow circle stands for number of downregulated DE genes between group M and group C. (e) GO annotation results of 53 DEGs in retro-WAT. (f) KEGG pathway for the 53 DEGs.

We compared the gene expression profiles of DIO rats with those of the EA-treated or untreated rats using Venn diagrams. The results revealed that EA treatment downregulated the expression of 610 genes, which were upregulated in group *M* (Figure 3(a)). Gene ontology (GO) annotation indicated that these genes were mainly expressed in the intracellular part, organelle, and cytoplasm, which were significantly enriched in several biological processes. They were functionally clustered into muscle contraction, muscle system process, and muscle cell development (Figure 3(b)). Kyoto Encyclopedia of Genes and Genomes (KEGG) pathway analysis confirmed that these downregulated DEGs belonged to the glycolysis/gluconeogenesis, glucagon signaling pathway, and peroxisome proliferator activated receptor (PPAR) signaling pathway (Figure 3(c)). However, the 53 upregulated genes in the hypothalamus were also mainly located in the extracellular region and extracellular space and were involved in the vitamin biosynthetic process (Figures 3(d) and 3(e)). Several enriched pathways were detected, including glycine, serine, and threonine metabolism, biosynthesis of amino acids, and nonalcoholic fatty liver disease (Figure 3(f)). To provide more detailed information, we listed the top 20 DGEs with the highest fold changes in retro-WAT in Supplementary Appendices 1 and 2.

#### 3.2.3. TE Treatment Reversed the Abnormal Gene Expression in DIO Rats

**Figure 4:**
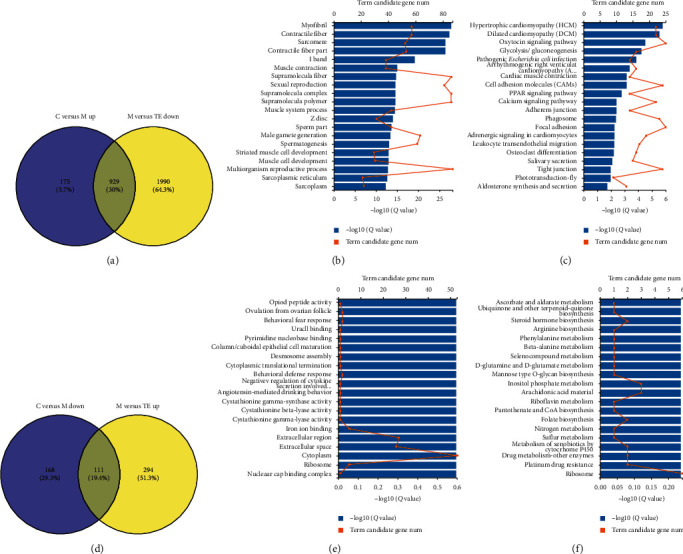
TE treatment reversed the abnormal gene expressions in DIO rats. (a) Purple circle represents the number of downregulated DE genes between group M and group TE; yellow circle stands for the number of upregulated DE genes between group M and group C. (b) GO annotation results of 929 DEGs in retro-WAT. (c) KEGG pathway for the 929 DEGs. (d) Purple circle represents the number of upregulated DE genes between group M and group TE; yellow circle stands for the number of downregulated DE genes between group M and group C. (e) GO annotation results of 111 DEGs in retro-WAT. (f) KEGG pathway for the 111 DEGs.

By comparing the upregulated genes of M vs. C and downregulated M vs. TE, 929 shared DE genes were identified (Figure 4(a)). The identified DEGs were further analyzed by GO term enrichment. The abundant DEGs were categorized into 20 major functional groups, and functional matrices were myofibril, contractile fiber, and sarcomere (Figure 4(b)). KEGG pathway enrichment was then performed to categorize the DEGs. The downregulated terms were as follows: oxytocin signaling pathway, glycolysis/gluconeogenesis, cell adhesion molecules (CAMs), PPAR signaling pathway, calcium signaling pathway, and so on (Figure 4(c)). Likewise, in the downregulated genes, 111 were also found in the upregulated gene list (305) (Figure 4(d)). GO analysis revealed that they participated in the behavioral defense response and negative regulation of cytokine secretion involved in the immune response (Figure 4(e)). KEGG analysis showed that they mainly participated in D-Glutamine and D-Glutamate metabolism and mannose type O-glycan biosynthesis (Figure 4(f)). To provide more detailed information, we listed the top 20 DGEs with the highest fold changes in retro-WAT in Supplementary Appendices 3 and 4.

#### 3.2.4. EA + TE Treatment Reversed the Abnormal Gene Expression in DIO Rats

**Figure 5:**
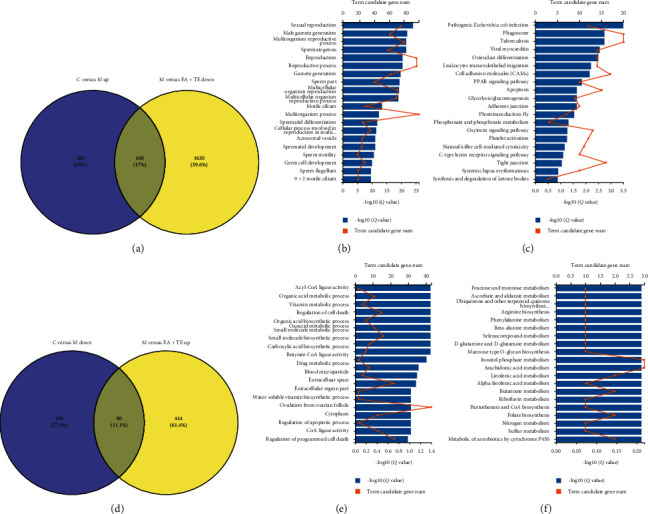
EA + TE treatment reversed the abnormal gene expressions in DIO rats. (a) Purple circle represents number of downregulated DE genes between group M and group EA + TE; yellow circle stands for number of upregulated DE genes between group M and group C. (b) GO annotation results of 640 DEGs in retro-WAT. (c) KEGG pathway for the 640 DEGs. (d) Purple circle represents number of upregulated DE genes between group M and group EA + TE; yellow circle stands for number of downregulated DE genes between group M and group C. (e) GO annotation results of 80 DEGs in retro-WAT. (f) KEGG pathway for the 80 DEGs.

To examine the overlap among the DEGs of each pairwise comparison, a Venn diagram was used to show the results. There were 640 common DE genes among the upregulated genes of M vs. C and downregulated genes of M vs. EA + TE (Figure 5(a)). We tested the enrichment of terms for the gene list and identified the significantly enriched GO terms, such as multiorganism process and reproduction (Figure 5(b)). Several KEGG pathways were commonly significantly enriched in 640 common DEGs, including cell adhesion molecules (CAMs), PPAR signaling pathway, apoptosis, glycolysis/gluconeogenesis, and synthesis and degradation of ketone bodies (Figure 5(c)). In the same way, we found that, in the 279 downregulated genes from the model group, 28.6% (80) of the genes were upregulated by EA + TE treatment (Figure 5(d)). In the GO enrichment analysis, several GO terms were significantly enriched, including acyl-CoA ligase activity, organic acid metabolic process, and vitamin metabolic process (Figure 5(e)). Pathway analysis was conducted for the DEGs.  Figure 5(f) shows the related pathways for the 80 overlapping genes, such as fructose and mannose metabolism, ascorbate and aldarate metabolism, and mannose type O-glycan biosynthesis. To provide more detailed information, we listed the top 20 DGEs with the highest fold changes in retro-WAT in Supplementary Appendices 5 and 6.

## 4. Discussion

As an effective alternative therapy, acupuncture has been used for the treatment of various kinds of diseases [22]. Electroacupuncture, a type of acupuncture, applies a pulsed current to the needle, which acts on the body's meridian and acupoints to treat diseases. EA is a combination of acupuncture and electrophysiological effects, which can improve the treatment effect and reduce the workload of manual twisting of the needle. EA has become a commonly used treatment method in clinical practice for obesity [14].

According to a previous report, acupuncture showed a beneficial effect on antiobesity [18]. A study [23] revealed that acupuncture can act on the satiety center of the ventromedial nucleus in the hypothalamus to enhance its electrical activity, upregulate the expression of obesity-related peptides in the hypothalamus, and reduce food intake, thereby reducing body mass. Acupuncture can reduce serum insulin levels in obese rats to improve insulin sensitivity [24]. In addition, EA is helpful for reducing the serum TC, triglyceride, and low-density lipoprotein concentrations in obese rats, thus exerting a benign regulatory effect on lipid metabolism [25]. It has also been found in another study [26] that EA can increase the expression of uncoupling protein 1 in adipose tissue and promote the browning of white fat. Therefore, acupuncture has the potential to cure diseases by causing many changes in gene expression. As a result, in this study, RNA-seq analysis was used to investigate the molecular mechanisms of EA in antiobesity from the whole gene expression aspect.

Our study used a high-fat diet-induced obesity model, which is convenient and stable and has been widely used [27]. High-fat food is the main environmental factor leading to simple obesity; therefore, this model simulates the physiological changes in obese patients. For acupoints selection, studies show that ST36 can regulate multiple systems, such as the digestive system, immune system, and endocrine system [28, 29]. In addition, ST36 is a classical acupoint for obesity treatment in experimental studies. Wang reported that Zusanli acupoint is used more frequently than others in animal models of simple obesity [18]. However, most studies used multiple points. The effect and molecular mechanism of acupuncture at a single acupoint in Zusanli need to be explored. Therefore, we selected ST36 to be stimulated by EA in this study.

In this study, we measured body weight and the ratio of retro-WAT to ascertain the antiobesity effect of EA and TE in DIO rats and explored the mechanism of EA and TE at the molecular level of the transcriptome in the WAT of DIO rats. Based on the results, the body weight of rats in the model group was significantly increased, suggesting that the model was successfully established. In EA and TE, both interventions significantly decreased body weight and the retro-WAT/body weight ratio, indicating antiobesity eﬀects on DIO rats. In addition, the therapeutic effect of EA plus TE was better than that of simple EA and simple TE.

According to DEG analysis, 79.8% of DEGs in retro-WAT were upregulated due to obesity, indicating that obesity plays a role in activating but not repressing gene expression in retro-WAT. EA treatment reversed the expression change in retro-WAT, and 47.3% of the upregulated genes were decreased to normal levels following EA treatment. These genes are mainly involved in catalyzing the regeneration of ATP (Ckm, Atp2a1), structural components of the cytoskeleton (Xirp2, Acta1), and muscle function (Myh4). Then, GO and KEGG pathway enrichment were performed to categorize the DEGs in comparing groups C vs. M and M vs. EA. In the GO enrichment analysis, several GO terms were commonly significantly enriched in the DEGs, mainly involved in myofibril, contractile fiber, muscle system process, and muscle cell development (Figures 3(b) and 3(e)). Further analysis revealed that EA treatment can reverse the changes in several KEGG pathways caused by obesity: PPAR signaling pathway, glycolysis/gluconeogenesis, calcium signaling pathway, and glucagon signaling pathway (Figures 3(c) and 3(f)). Changing these KEGG pathways can be considered as another mechanism of EA towards obesity.

As for TE treatment, it could reverse the expression change of 929 (30%) upregulated DEGs induced by obesity. Surprisingly, several genes were listed both in the top 20 EA-dependent downregulated DEGs in retro-WAT and in the top 20 TE-dependent downregulated DEGs in retro-WAT (e.g., Myh4, Ttn, Ckm, Xirp2, Odf1, Smcp, Atp2a1, Acta1, and Odf1). In addition, several GO terms were commonly significantly enriched in both M vs. TE and M vs. EA, such as myofibril and muscle contraction (Figures 4(b) and 4(e)). Compared with the model group, the pathological genes regulated by TE include relatively more functional pathways, such as cell adhesion molecules (CAMs), PPAR signaling pathway, calcium signaling pathway, aldosterone synthesis, and secretion (Figures 4(c) and 4(f)). The results show that there are many similarities in the molecular mechanism of EA and exercise to weight loss (Supplementary Appendices 2 and 4).

## 5. Conclusion

In summary, our study indicates that EA treatment at Zusanli (ST36) and TE treatment exerted effects of weight loss on DIO rats and reversed the obesity-related phenotypes by modulating abnormal gene profiles in the retro-WAT. Our results suggest that the antiobesity effect of EA and TE may be achieved by pursuing multiple targets, which provide a clue for further investigation into the molecular mechanisms. We will provide more experimental evidence for the antiobesity effect of acupuncture through further exploration in our next study.

## Figures and Tables

**Table 1:** Quality control of RNA sample.

Sample	RIN/RQN	28S/18S	Conc (ng/uL)
Control (1)	8.2	1.5	109
Control (2)	8.2	1.4	56
Control (3)	7.8	1.2	92
Model (1)	8.5	1.6	68
Model (2)	8.2	1.3	115
Model (3)	8.0	1.3	290
EA (1)	8.4	1.5	56
EA (2)	8.4	1.2	107
EA (3)	8.1	1.5	187
TE (1)	8.7	1.7	126
TE (2)	8.6	1.3	184
TE (3)	8.6	1.4	188
EA + TE (1)	8.2	1.4	174
EA + TE (2)	8.5	1.4	193
EA + TE (3)	8.5	1.5	82

**Table 2:** Differentially expressed genes (DEGs) with a log2 (FC) > |±1| and *p* < 0.05.

DEGs	C vs. M	M vs. EA	M vs. TE	M vs. EA + TE
Upregulated	1104 (79.8%)	117 (12.8%)	405 (12.1%)	524 (18.7%)
Downregulated	279 (20.2%)	796 (87.2%)	2919 (81.9%)	2270 (81.7%)
Total	1383 (100%)	913 (100%)	3324 (100%)	2794 (100%)

## Data Availability

All data during the study are available from the corresponding author upon request.

## Supplementary Materials

Supplementary Materials Appendix 1: top 20 EA-dependent upregulated DEGs in retro-WAT. Appendix 2: top 20 EA-dependent downregulated DEGs in retro-WAT. Appendix 3: top 20 TE-dependent upregulated DEGs in retro-WAT. Appendix 4: top 20 TE-dependent downregulated DEGs in retro-WAT. Appendix 5: top 20 EA + TE-dependent upregulated DEGs in retro-WAT. Appendix 6: top 20 EA + TE-dependent downregulated DEGs in retro-WAT.
