# Supplementary material for: Genome-Wide Regulation of Electroacupuncture and Treadmill Exercise on Diet-Induced Obese Rats
Source: Evid Based Complement Alternat Med. 2020 Sep 24;2020:8764507. doi: 10.1155/2020/8764507 (PMC7533018; doi:10.1155/2020/8764507)
Supplement: Supplementary Materials — Appendix 1: top 20 EA-dependent upregulated DEGs in retro-WAT. Appendix 2: top 20 EA-dependent downregulated DEGs in retro-WAT. Appendix 3: top 20 TE-dependent upregulated DEGs in retro-WAT. Appendix 4: top 20 TE-dependent downregulated DEGs in retro-WAT. Appendix 5: top 20 EA + TE-dependent upregulated DEGs in retro-WAT. Appendix 6: top 20 EA + TE-dependent downregulated DEGs in retro-WAT. [file 8764507.f1.doc]

**Appendix 1** Top 20 EA-dependent upregulated DEGs in retro-WAT

| Gene name | Description | FPKM | | | Log2 (fold change) | |
| --- | --- | --- | --- | --- | --- | --- |
|  |  | Con | Model | EA | Con vs Model | Model vs EA |
| Alkbh6 | alkB homolog 6 | 14.5 | 0.02 | 9.95 | -9.28 | 8.73 |
| LOC108349606 | 60S ribosomal protein L7a-like | 0.48 | 0.00 | 3.01 | -5.24 | 7.91 |
| LOC102550456 | TSC22 domain family protein 4-like | 0.57 | 0.00 | 0.56 | -7.14 | 7.14 |
| LOC102550530 | MARCKS-related protein-like | 10.55 | 0.00 | 0.95 | -10.48 | 7.03 |
| LOC100909795 | colorectal mutant cancer protein-like | 2.11 | 0.00 | 0.16 | -10.54 | 6.83 |
| LOC100911077 | uncharacterized LOC100911077 | 1.41 | 0.00 | 1.29 | -6.73 | 6.62 |
| Cth | cystathionine gamma-lyase | 0.36 | 0.00 | 0.28 | -6.01 | 5.66 |
| LOC100911130 | mitochondrial import inner membrane translocase subunit Tim17-B-like | 0.46 | 0.00 | 0.45 | -5.35 | 5.35 |
| LOC102548576 | uncharacterized LOC102548576 | 0.64 | 0.03 | 0.66 | -4.76 | 4.79 |
| LOC102546359 | uncharacterized LOC102546359 | 0.17 | 0.00 | 0.18 | -4.58 | 4.70 |
| Nat8b | N-acetyltransferase 8B | 3.85 | 0.21 | 3.07 | -4.19 | 3.87 |
| Afp | alpha-fetoprotein | 0.50 | 0.03 | 0.37 | -4.09 | 3.67 |
| LOC108348184 | pyridoxal-dependent decarboxylase domain-containing protein 1-like | 1.35 | 0.07 | 0.81 | -4.18 | 3.45 |
| LOC102555134 | uncharacterized LOC102555134 | 0.65 | 0.09 | 0.75 | -2.95 | 3.15 |
| LOC108348085 | beta-1,4-glucuronyltransferase 1 | 1.94 | 0.32 | 2.30 | -2.59 | 2.81 |
| Alb | albumin | 112.12 | 29.87 | 176.49 | -1.90 | 2.57 |
| LOC108348948 | uncharacterized LOC108348948 | 0.12 | 0.02 | 0.13 | -2.37 | 2.51 |
| Naa11 | N(alpha)-acetyltransferase 11, NatA catalytic subunit | 0.16 | 0.04 | 0.2 | -1.85 | 2.32 |
| Ubd | ubiquitin D | 4.11 | 1.23 | 5.46 | -1.73 | 2.15 |
| Lix1 | limb and CNS expressed 1 | 1.19 | 0.21 | 0.91 | -2.53 | 2.15 |

**Appendix 2 Top 20 EA-dependent downregulated DEGs in retro-WAT**

| Gene name | Description | FPKM | | | Log2 (fold change) | |
| --- | --- | --- | --- | --- | --- | --- |
|  |  | Con | Model | EA | Con vs Model | Model vs EA |
| Myh4 | myosin heavy chain 4 | 0.01 | 89.64 | 0.01 | 13.47 | -12.79 |
| Ttn | titin | 0.00 | 0.61 | 0.00 | 12.71 | -12.71 |
| Ckm | creatine kinase, M-type | 0.12 | 322.27 | 0.08 | 11.53 | -11.94 |
| Odf1 | outer dense fiber of sperm tails 1 | 0.04 | 15.96 | 0.00 | 8.59 | -10.59 |
| Xirp2 | xin actin-binding repeat containing 2 | 0.00 | 0.87 | 0.00 | 10.10 | -10.10 |
| LOC100909761 | myotilin-like | 0.00 | 4.69 | 0.00 | 9.99 | -9.99 |
| Ndufa10l1 | NADH dehydrogenase (ubiquinone) 1 alpha subcomplex 10-like 1 | 2.96 | 6.56 | 0.00 | 1.16 | -9.91 |
| Atp2a1 | ATPase sarcoplasmic/endoplasmic reticulum Ca2+ transporting 1 | 0.12 | 117.60 | 0.13 | 9.97 | -9.84 |
| Tnp2 | transition protein 2 | 0.00 | 28.89 | 0.04 | 10.61 | -9.61 |
| Tnnc2 | troponin C2, fast skeletal type | 0.30 | 164.07 | 0.22 | 9.09 | -9.55 |
| Acta1 | actin, alpha 1, skeletal muscle | 0.26 | 424.31 | 0.64 | 10.68 | -9.36 |
| Rps18l1 | ribosomal protein S18-like 1 | 0.00 | 1.38 | 0.00 | 9.53 | -9.26 |
| Smcp | sperm mitochondria-associated cysteine-rich protein | 0.00 | 12.54 | 0.02 | 10.07 | -9.07 |
| Prm1 | protamine 1 | 0.00 | 37.92 | 0.00 | 8.87 | -8.87 |
| Tnni2 | troponin I2, fast skeletal type | 0.21 | 84.44 | 0.21 | 8.66 | -8.66 |
| Tcp10b | t-complex protein 10b | 0.00 | 1.55 | 0.00 | 8.27 | -8.27 |
| Art1 | ADP-ribosyltransferase 1 | 0.00 | 4.42 | 0.01 | 9.27 | -8.27 |
| NEWGENE_1308612 | MRG/MORF4L binding protein | 0.85 | 1.96 | 0.00 | 1.23 | -8.25 |
| Actn3 | actinin alpha 3 | 0.23 | 63.95 | 0.23 | 8.10 | -8.14 |
| Cmtm2a | CKLF-like MARVEL transmembrane domain containing 2A | 0.00 | 2.81 | 0.00 | 8.10 | -8.10 |

**Appendix 3 Top 20 TE-dependent upregulated DEGs in retro-WAT**

| Gene name | Description | FPKM | | | Log2 (fold change) | |
| --- | --- | --- | --- | --- | --- | --- |
|  |  | Con | Model | TE | Con vs Model | Model vs TE |
| LOC102550530 | MARCKS-related protein-like | 10.55 | 0.00 | 12.25 | -10.48 | 10.70 |
| LOC100911615 | patatin-like phospholipase domain-containing protein 2-like | 2.30 | 0.00 | 2.27 | -9.05 | 9.06 |
| Alkbh6 | alkB homolog 6 | 14.53 | 0.02 | 11.15 | -9.28 | 8.90 |
| LOC100910446 | syntaxin-7-like | 5.39 | 0.00 | 1.49 | -10.09 | 8.24 |
| Cth | cystathionine gamma-lyase | 0.36 | 0.00 | 0.80 | -6.01 | 7.16 |
| LOC108349606 | 60S ribosomal protein L7a-like | 0.48 | 0.00 | 0.83 | -5.24 | 6.05 |
| LOC108352750 | toll-like receptor 6 | 0.24 | 0.00 | 0.34 | -4.86 | 5.42 |
| LOC102548576 | uncharacterized LOC102548576 | 0.64 | 0.03 | 0.83 | -4.76 | 5.12 |
| Gprin3 | GPRIN family member 3 | 4.52 | 0.89 | 25.94 | -2.36 | 4.87 |
| LOC100363469 | ribosomal protein S24-like | 0.66 | 0.00 | 0.43 | -5.03 | 4.43 |
| Afp | alpha-fetoprotein | 0.50 | 0.03 | 0.58 | -4.09 | 4.31 |
| LOC102551541 | uncharacterized LOC102551541 | 0.13 | 0.02 | 0.39 | -2.47 | 4.10 |
| LOC100911313 | regulator of microtubule dynamics protein 3-like | 4.24 | 0.24 | 3.85 | -4.13 | 4.01 |
| Nat8b | N-acetyltransferase 8B | 3.85 | 0.21 | 3.36 | -4.19 | 3.99 |
| Lix1 | limb and CNS expressed 1 | 1.19 | 0.21 | 3.16 | -2.53 | 3.95 |
| LOC102556347 | carbonyl reductase [NADPH] 1-like | 26.50 | 4.48 | 56.04 | -2.58 | 3.66 |
| LOC102553010 | leukocyte elastase inhibitor A-like | 2.79 | 0.79 | 9.70 | -1.82 | 3.63 |
| LOC102550339 | uncharacterized LOC102550339 | 0.10 | 0.01 | 0.01 | -4.17 | 3.59 |
| Dppa4 | developmental pluripotency associated 4 | 0.41 | 0.03 | 0.29 | -3.78 | 3.29 |
| LOC108350691 | uncharacterized LOC108350691 | 18.09 | 1.21 | 10.39 | -3.90 | 3.11 |

**Appendix 4** Top 20 TE-dependent downregulated DEGs in retro-WAT

| Gene name | Description | FPKM | | | Log2 (fold change) | |
| --- | --- | --- | --- | --- | --- | --- |
|  |  | Con | Model | TE | Con vs Model | Model vs TE |
| Ttn | titin | 0.00 | 0.61 | 0.00 | 12.71 | -12.70 |
| Myh4 | myosin heavy chain 4 | 0.01 | 89.64 | 0.03 | 13.47 | -11.61 |
| Ckm | creatine kinase, M-type | 0.12 | 322.27 | 0.19 | 11.53 | -10.71 |
| Xirp2 | xin actin-binding repeat containing 2 | 0.00 | 0.87 | 0.00 | 10.10 | -10.10 |
| Smcp | sperm mitochondria-associated cysteine-rich protein | 0.00 | 12.54 | 0.00 | 10.07 | -10.07 |
| Atp2a1 | ATPase sarcoplasmic/endoplasmic reticulum Ca2+ transporting 1 | 0.12 | 117.60 | 0.11 | 9.97 | -10.04 |
| LOC100909761 | myotilin-like | 0.00 | 4.69 | 0.00 | 9.99 | -9.98 |
| Tnp1 | transition protein 1 | 0.00 | 21.94 | 0.00 | 9.84 | -9.83 |
| Acta1 | actin, alpha 1, skeletal muscle | 0.26 | 424.31 | 0.48 | 10.68 | -9.79 |
| Oaz3 | ornithine decarboxylase antizyme 3 | 0.28 | 20.21 | 0.02 | 6.20 | -9.78 |
| Odf1 | outer dense fiber of sperm tails 1 | 0.04 | 15.96 | 0.02 | 8.59 | -9.59 |
| Crisp2 | cysteine-rich secretory protein 2 | 0.01 | 5.07 | 0.00 | 8.51 | -9.51 |
| NEWGENE_1310680 | transmembrane protein 151A | 0.66 | 2.52 | 0.00 | 1.96 | -9.41 |
| LOC100361476 | vasculin-like protein 1-like | 0.04 | 2.03 | 0.00 | 5.53 | -9.33 |
| Igfn1 | immunoglobulin-like and fibronectin type III domain containing 1 | 0.00 | 0.83 | 0.00 | 9.14 | -9.13 |
| Trim54 | tripartite motif-containing 54 | 0.03 | 3.67 | 0.00 | 7.08 | -9.08 |
| LOC100271845 | hypothetical protein LOC100271845 | 0.00 | 2.11 | 0.00 | 9.01 | -9.01 |
| Spata18 | spermatogenesis associated 18 | 0.02 | 2.52 | 0.00 | 6.99 | -8.99 |
| Tcp11 | t-complex 11 | 0.03 | 2.68 | 0.00 | 6.39 | -8.97 |
| Actn3 | actinin alpha 3 | 0.23 | 63.95 | 0.13 | 8.10 | -8.90 |

**Appendix 5** Top 20 EA + TE-dependent upregulated DEGs in retro-WAT

| Gene name | Description | FPKM | | | Log2 (fold change) | |
| --- | --- | --- | --- | --- | --- | --- |
|  |  | Con | Model | EA+TE | Con vs Model | Model vs EA+TE |
| LOC100911615 | patatin-like phospholipase domain-containing protein 2-like | 2.30 | 0.00 | 2.27 | -9.05 | 10.64 |
| LOC102550530 | MARCKS-related protein-like | 10.55 | 0.00 | 11.52 | -10.48 | 10.62 |
| Alkbh6 | alkB homolog 6 | 14.53 | 0.02 | 9.91 | -9.28 | 8.73 |
| LOC100910446 | syntaxin-7-like | 5.39 | 0.00 | 1.29 | -10.09 | 8.04 |
| LOC100909795 | colorectal mutant cancer protein-like | 2.11 | 0.00 | 0.32 | -10.54 | 7.84 |
| LOC100911725 | 6-phosphofructo-2-kinase/fructose-2,6-bisphosphatase 4-like | 0.25 | 0.00 | 0.60 | -6.30 | 7.57 |
| LOC102550456 | TSC22 domain family protein 4-like | 0.00 | 0.57 | 0.56 | -7.14 | 7.13 |
| LOC100911077 | uncharacterized LOC100911077 | 1.41 | 0.00 | 1.38 | -6.73 | 6.72 |
| Cth | cystathionine gamma-lyase | 0.36 | 0.00 | 0.50 | -6.01 | 6.51 |
| LOC103692976 | cyclin-T1-like | 0.13 | 0.00 | 0.08 | -6.53 | 5.89 |
| LOC691249 | similar to casein kinase 1, gamma 3 | 0.213 | 0.00 | 0.11 | -6.31 | 5.40 |
| LOC100911130 | mitochondrial import inner membrane translocase subunit Tim17-B-like | 0.46 | 0.00 | 0.44 | -5.35 | 5.34 |
| LOC102552104 | MLV-related proviral Env polyprotein-like | 0.36 | 0.00 | 0.20 | -6.15 | 5.23 |
| LOC102548576 | uncharacterized LOC102548576 | 0.64 | 0.03 | 0.63 | -4.76 | 4.72 |
| Cyp2e1 | cytochrome P450, family 2, subfamily e, polypeptide 1 | 6.70 | 0.85 | 14.26 | -2.99 | 4.07 |
| Afp | alpha-fetoprotein | 0.50 | 0.03 | 0.41 | -4.09 | 3.83 |
| Nat8b | N-acetyltransferase 8B | 3.85 | 0.21 | 2.52 | -4.19 | 3.58 |
| Gprin3 | GPRIN family member 3 | 4.52 | 0.89 | 9.83 | -2.36 | 3.48 |
| Ubd | ubiquitin D | 4.11 | 1.23 | 11.93 | -1.73 | 3.29 |
| Rpl9 | ribosomal protein L9 | 15.25 | 1.09 | 9.91 | -3.78 | 3.17 |

**Appendix 6** Top 20 EA + TE-dependent downregulated DEGs in retro-WAT

| Gene name | Description | FPKM | | | Log2 (fold change) | |
| --- | --- | --- | --- | --- | --- | --- |
|  |  | Con | Model | EA+TE | Con vs Model | Model vs EA+TE |
| Smcp | sperm mitochondria-associated cysteine-rich protein | 0.00 | 12.54 | 0.00 | 10.07 | -10.09 |
| Tnp1 | transition protein 1 | 0.00 | 21.94 | 0.03 | 9.84 | -9.85 |
| Oaz3 | ornithine decarboxylase antizyme 3 | 0.28 | 20.21 | 0.02 | 6.20 | -9.80 |
| Tnp2 | transition protein 2 | 0.00 | 28.89 | 0.11 | 10.61 | -9.62 |
| Odf1 | outer dense fiber of sperm tails 1 | 0.04 | 15.96 | 0.02 | 8.59 | -9.61 |
| Crisp2 | cysteine-rich secretory protein 2 | 0.01 | 5.07 | 0.00 | 8.51 | -9.53 |
| NEWGENE_1310680 | transmembrane protein 151A | 0.66 | 2.52 | 0.00 | 1.96 | -9.43 |
| LOC100271845 | hypothetical protein LOC100271845 | 0.00 | 2.11 | 0.00 | 9.01 | -9.03 |
| Spata18 | spermatogenesis associated 18 | 0.02 | 2.52 | 0.00 | 6.99 | -9.01 |
| Tcp11 | t-complex 11 | 0.03 | 2.68 | 0.00 | 6.39 | -8.99 |
| Prm1 | protamine 1 | 0.00 | 37.92 | 0.00 | 8.87 | -8.89 |
| Akap4 | A-kinase anchoring protein 4 | 0.00 | 1.45 | 0.00 | 8.68 | -8.70 |
| Tcp10b | t-complex protein 10b | 0.00 | 1.55 | 0.00 | 8.27 | -8.29 |
| Cmtm2a | CKLF-like MARVEL transmembrane domain containing 2A | 0.04 | 2.81 | 0.00 | 8.10 | -8.12 |
| Nt5c1b | 5'-nucleotidase, cytosolic IB | 0.03 | 1.48 | 0.00 | 5.52 | -8.12 |
| Adam3a | ADAM metallopeptidase domain 3A | 0.00 | 1.06 | 0.00 | 8.07 | -8.09 |
| Iqcf3 | IQ motif containing F3 | 0.00 | 2.35 | 0.00 | 8.05 | -8.07 |
| LOC108349760 | dentin sialophosphoprotein-like | 0.02 | 0.65 | 0.00 | 5.32 | -7.92 |
| LOC103690996 | uncharacterized LOC103690996 | 0.00 | 5.11 | 0.00 | 7.81 | -7.82 |
| NEWGENE_621802 | calcium/calmodulin-dependent protein kinase II gamma | 0.00 | 0.60 | 0.00 | 7.77 | -7.79 |
